# Millennial processes of population decline, range contraction and near extinction of the European bison

**DOI:** 10.1098/rspb.2023.1095

**Published:** 2023-12-13

**Authors:** July A. Pilowsky, Stuart C. Brown, Bastien Llamas, Ayla L. van Loenen, Rafał Kowalczyk, Emilia Hofman-Kamińska, Ninna H. Manaseryan, Viorelia Rusu, Matija Križnar, Carsten Rahbek, Damien A. Fordham

**Affiliations:** ^1^ The Environment Institute and School of Biological Sciences, University of Adelaide, South Australia 5005, Australia; ^2^ Australian Centre for Ancient DNA, School of Biological Sciences, University of Adelaide, South Australia 5005, Australia; ^3^ ARC Centre of Excellence for Australian Biodiversity and Heritage, University of Adelaide, South Australia 5005, Australia; ^4^ Center for Macroecology, Evolution and Climate, Globe Institute, University of Copenhagen, Copenhagen Ø 2100, Denmark; ^5^ Center for Mountain Biodiversity, Globe Institute, University of Copenhagen, Copenhagen Ø 2100, Denmark; ^6^ Section for Evolutionary Genomics, Globe Institute, University of Copenhagen, Copenhagen K 1350, Denmark; ^7^ Indigenous Genomics Research Group, Telethon Kids Institute, Adelaide, South Australia 5001, Australia; ^8^ National Centre for Indigenous Genomics, Australian National University, Canberra, Australian Capital Territory 2601, Australia; ^9^ Mammal Research Institute, Polish Academy of Sciences, 17-230 Białowieża, Poland; ^10^ The Scientific Centre of Zoology and Hydroecology of National Academy of Sciences of Armenia, Institute of Zoology, 0014 Yerevan, Republic of Armenia; ^11^ Institute of Zoology, Academy of Sciences of Moldova, Chisinau MD-2028, Republic of Moldova; ^12^ Slovenian Museum of Natural History, Department of Geology, SI-1001 Ljubljana, Slovenia; ^13^ Danish Institute for Advanced Study, University of Southern Denmark, Odense M 5230, Denmark; ^14^ Institute of Ecology, Peking University, Beijing, People's Republic of China

**Keywords:** extinction dynamics, rewilding, process-based model, megafauna, range dynamics, conservation biogeography

## Abstract

European bison (*Bison bonasus*) were widespread throughout Europe during the late Pleistocene. However, the contributions of environmental change and humans to their near extinction have never been resolved. Using process-explicit models, fossils and ancient DNA, we disentangle the combinations of threatening processes that drove population declines and regional extinctions of European bison through space and across time. We show that the population size of European bison declined abruptly at the termination of the Pleistocene in response to rapid environmental change, hunting by humans and their interaction. Human activities prevented populations of European bison from rebounding in the Holocene, despite improved environmental conditions. Hunting caused range loss in the north and east of its distribution, while land use change was responsible for losses in the west and south. Advances in hunting technologies from 1500 CE were needed to simulate low abundances observed in 1870 CE. While our findings show that humans were an important driver of the extinction of the European bison in the wild, vast areas of its range vanished during the Pleistocene–Holocene transition because of post-glacial environmental change. These areas of its former range have been climatically unsuitable for millennia and should not be considered in reintroduction efforts.

## 1. Introduction

The European bison (*Bison bonasus*) is a large grazer that was once widely distributed in Europe and western Asia [1]. During the Pleistocene–Holocene transition, the population and geographical range of the European bison collapsed [2], with the species going extinct in the wild in 1927 [3]. Since its near extinction, enormous effort and resources have been directed towards restoring healthy wild populations of European bison from captive survivors [3]. These conservation measures have been incredibly effective, resulting in a progressive downgrading of extinction risk for European bison, from extinct in the wild in the early twentieth century to near threatened in less than 100 years [4].

As European bison increase in number and range size [4], their long-term persistence relies on knowing how and why they nearly went extinct in the first place. Currently, there is much speculation as to whether the pathway to near extinction for the European bison was a recent and abrupt human-driven event, or a much longer and more drawn-out process that involved past climatic and environmental change as well as sustained hunting by humans [5]. A better biogeographic understanding of the ecological processes of population decline and range contraction for European bison is needed to inform evidence-based solutions for its future protection and recovery [6,7].

It has been hypothesized that the cause of the European bison's near extinction was habitat loss due to deforestation, based on an assumption that the European bison is a forest specialist [2]. However, this hypothesis is based on a relatively short temporal perspective, founded on historical records and fossil evidence from the Holocene, when Europe was already mostly forested [8]. A competing theory is that the European bison is not a forest specialist but primarily a grazer, adapted to mosaic rather than strictly forest habitats [5]. This is supported by stable isotope data from modern and ancient European bison bone collagen, which show a shift in habitat use from open to closed habitats in the early to late Holocene—a response driven by wide-scale forest expansion [9]. According to this theory, the European bison became trapped in expanding suboptimal forest habitats as Eurasia emerged from the last ice age [10]. At this time, human populations that hunted European bison for food and skins [2] were spreading north and becoming more ubiquitous across Eurasia [11]. Hence, it is argued that hunting by humans, particularly in more populated open habitats, further amplified the climate-driven retreat of European bison to forested habitats [12]. A third view is that human impacts alone were the primary cause of the range collapse and population decline of Eurasian megafauna, including the European bison [13,14]. What is clear from these competing theories is that the contributions of environmental change, humans and their interactions on the scale, rate and geographical patterning of loss of the European bison is still unclear.

Currently, there are approximately 7300 free-ranging European bison [15]. Efforts to re-establish and conserve the species in the wild are far-reaching. This is because the European bison is an ecosystem engineer with a key role in maintaining landscapes and biodiversity [16]. By debarking trees and browsing on tree seedlings, the European bison restores threatened grassland habitats, preventing forest encroachment [17]. Despite these important ecosystem services, a poorly resolved understanding of the biogeography of the European bison has meant that its rewilding has been done without a strong understanding of habitats and regions where it once thrived [5,18]. Consequently, European bison have been released at sites ranging from the coastal dunes of the Netherlands [16] to the mountains of the French Alps [19], with mixed success [4]. Thus, continued reintroduction efforts to recover the European bison and restore grasslands will benefit from a more thorough understanding of its past range dynamics, including the causes of its range collapse and population decline, which, depending on the hypothesis, occurred many centuries to millennia ago.

While correlative ecological niche models (ENMs) have provided approximations of the range of the European bison in the Holocene [20], these projections did not capture (i) the full breadth of environmental conditions that the European bison is likely to have occupied and thrived in prior to the Holocene, and (ii) the complexity of drivers that caused population decline and range contraction during this period [5]. Recent developments in process-driven ecological models are providing more robust projections of the past range and extinction dynamics of megafauna, including for species that the European bison coexisted with during the late Pleistocene [21]. These projections are being made using spatially explicit population models (SEPMs) that explicitly simulate functions of global change, ecological processes and their interactions on the structure and dynamics of geographical ranges of species [22]. Results from these simulation studies—validated with real-world data—have shown that pathways to range collapse and extinction are long and lasting [6], and that species respond demographically to biotic and abiotic stressors that operate at local-to-regional scales, varying through time [23].

Here, we detect and decipher the potential drivers and ecological processes responsible for the near extinction of the European bison using a detailed validated simulation approach that integrates high-resolution reconstructions of past climates and densities of people, and inferences of demographic change from fossils, historical records and ancient DNA. This statistical simulation approach uses new radiocarbon-dated fossils of European bison, representing some of the oldest ever found. Our 21 000-year reconstructions of the structure and dynamics of the geographical range of the European bison reveal important interactions between climatic warming and human pressures that drove the timing, magnitude, and pattern of range contraction and population decline of the European bison. They also show where the European bison would be distributed today if hunting and land use change had not occurred, providing much needed information for the future management of this species.

## 2. Methods

We built 55 000 SEPMs that simulate interactions between the metapopulation dynamics of European bison, environmental variability, human hunting and land use change [23]. We used these models to reconstruct 21 000 years of European bison range dynamics across Eurasia. We validated model projections of spatio-temporal abundance using pattern-oriented modelling (POM) methods [24], drawing on inferences of demographic change from historical accounts and 120 radiocarbon-dated fossils. The latter included 14 previously unpublished radiocarbon dates [25]. Models were coded in R v 4.2.0 using the package paleopop [26].

### (a) Ecological niche

To reconstruct the ecological niche of the European bison, we intersected radiocarbon (^14^C) dated and georeferenced fossils with simulated climate and land use projections. To do this, we compiled a database of ^14^C dated European bison fossils using published and unpublished sources [25]. Radiocarbon dating of all new fossil material (*n* = 14) was done using accelerator mass spectrometry at the Laboratory of Ion Beam Physics, Eidgenössische Technische Hochschule Zürich, Switzerland [25]. No ultra-filtration pre-treatment was needed, as the quality of the bone collagen had been tested before accelerator mass spectrometry to verify collagen yield, C:N proportion, %N in collagen and %C in collagen. Dated fossils without geolocations were geocoded manually using the name of the fossil site. The quality and reliability of all radiocarbon dates was assessed based on dating method, stratigraphy, association and material [27]. Only fossils with an age quality score greater than 10 were used [28]. This resulted in 120 high-quality ^14^C dates. The ^14^C ages of these fossils were calibrated using the OxCal tool [29] and the IntCal13 curve [30].

Occurrence records from fossils were intersected with monthly palaeoclimate data from the HadCM3 general circulation model (GCM), downscaled to 0.5° × 0.5° spatial resolution and bias-corrected to current-day conditions [31]. The HadCM3 GCM has previously been shown to accurately represent land and sea surface temperatures, precipitation and ocean circulation [32]. We extracted monthly data for annual precipitation, winter temperature and spring and summer evapotranspiration (electronic supplementary material, methods). These three climatic variables have been used previously to model the range dynamics of large vertebrates in Eurasia, including during the Pleistocene–Holocene transition [6,28]. These climatic variables limit forage availability in winter and forage nutritional constraints in summer [3]. Monthly climate data were temporally averaged over a 10-year period (the generation length of the European bison; see below) using a 30-year sliding window [21]. Climate data were projected to an Albers equal area projection centred on a reference latitude of 57.5°N and a reference longitude of 25°E, resulting in a grid-cell resolution of 86.6 by 75.6 km.

We used a dynamic vegetation model (LPJ-GUESS) [8] to determine habitat and resource availability for European bison. This model is coupled to HadCM3 palaeoclimate data but does not include herbivore feedbacks on plant structure or biomass, which is still debated [33]. We estimated the combined biomass of preferred plant functional types and adjusted these spatio-temporal estimates of biomass according to land use, using Hyde 3.2 [34]. Projections of adjusted biomass, representing human-driven vegetation change for European bison, were converted to the same Albers equal area projection as the climate data and temporally downscaled to a decadal time step using linear interpolation [21]. See electronic supplementary material, Methods for more details.

To model the ecological preferences of European bison spatio-temporally, we built a four-dimensional multi-temporal ENM [35]. To do this, we intersected the location, calibrated ^14^C date and its associated uncertainty (±2 s.d.) [36] of fossil occurrences with the climate variables and adjusted biomass. We removed any duplicate data created by two fossil occurrences falling within identical or overlapping spatio-temporal bins [21]. We used this dataset to create a Gaussian hypervolume of ecological suitability, a technique for niche estimation that does not require absence data [37]. We tuned the kernel density estimation bandwidth by optimizing the mean square error using cross-validation [37]. The resulting hypervolume was exhaustively subsampled to generate thousands of plausible realized niches [21,35] using niche marginality and volume [6,21]. Habitat suitability was projected into geographical space at 10-year intervals (the generational length of the European bison; see below). Habitat suitability scores were scaled between zero and one for each projection based on the 95th percentile of suitability for all projections [21]. See electronic supplementary material, methods for more details.

### (b) Human density

The population growth and expansion of Palaeolithic humans across Eurasia following the last glacial maximum (LGM, a period from 26.5 to 19 ka BP [38]) was modelled using a process-explicit climate-informed spatial genetic model (CISGeM) that accurately reconstructs global genetic patterns and arrival times of anatomically modern humans [39]. This model has previously been used to disentangle the impact of humans on megafauna over palaeo timescales [6,28]. CISGeM simulates effective population size (N_e_) using a cellular demographic model in which local N_e_ is a function of sea level, net primary productivity and local demography [40]. We ran CISGeM from 120 ka BP to present using climate data from the HadCM3 GCM [32]. To account for parameter uncertainty in projections of N_e_, we used published upper and lower confidence bounds for CISGeM parameters [40] to generate 4950 equally plausible unique models of human population growth and migration. We did this using Latin hypercube sampling [41].

We calculated the mean and standard deviation for N_e_ in each grid cell at each 25-year time step from 21 ka BP, then reprojected the values to the Albers equal area projection described above. N_e_ values were scaled between zero and one using the 95th percentile as an upper threshold, and used as a proxy for relative abundance [6]. We linearly interpolated the outputs from 25-year to 10-year time steps to match the ENM projections. We then generated plausible reconstructions of relative human abundance by sampling within ± 1 s.d. of N_e_ from a lognormal distribution. The centre of the sampling window within ± 1 s.d. of mean N_e_ was a variable model parameter in our European bison-climate-human process-explicit model [28]. The approach assumes that N_e_ and population abundance are positively related [42], which is generally true [43].

### (c) Process-explicit model

We used spatio-temporal estimates of habitat suitability and relative human abundance (from our ENMs and CISGeM, respectively) as inputs to a SEPM that simulated landscape-level population processes for the European bison, including metapopulation and dispersal dynamics [21,28]. Each grid cell was modelled with a scalar-type stochastic model that simulates the finite rate of population increase ‘R’, its variance and the population carrying capacity [44]. The approach has been used to skilfully reconstruct inferences of past range dynamics of large-bodied mammals [6], including a closely related species of bison (*Bison priscus*) [28]. The SEPM was run at generational time steps from 21 ka BP to 100 BP. A generation length of ten years was set based on the difference between reproductive lifespan and age at first birth [45].

Maximum annual growth rate and its variance were estimated using time-series data (see electronic supplementary material, methods). Population growth was modelled with a Ricker logistic density dependence function [46], with carrying capacity regulated by the habitat suitability in a given grid cell [28]. At a habitat suitability of 1, the carrying capacity was equal to the maximum density (electronic supplementary material, table S1), reducing with lower habitat suitability [47]. A negative Allee effect was simulated using an abundance threshold below which populations became locally extinct [21]. We estimated that between 5% and 25% of populations of European bison disperse per generation, with a maximum dispersal distance of 0–300 km (electronic supplementary material, table S1) [3]. A dispersal friction landscape [48] based on ice sheet reconstructions [49] and land use change (see §2a above) was used to ensure that bison only dispersed through ice-free grid cells, and that their dispersal was lower in urban, agricultural or pastoral environments. Harvesting was modelled as a nonlinear function of prey density, human density, hunting rate and prey availability [6,28]. These model parameters are described in more detail in the electronic supplementary material, methods.

Models centred on best estimates of demographic processes (population growth rate, dispersal, Allee effect), ecological attributes (niche volume and climatic specialization) and human hunting pressure (human abundance and hunting function) were varied across wide but plausible ranges (electronic supplementary material, table S1) using Latin hypercube sampling of uniform probability distributions [41]. This resulted initially in 25 000 model parametrizations, each with a different combination of values for demography, environmental preferences and exploitation by humans. Each model was run for a single replicate and validated using POM methods [21].

### (d) Model validation

We used POM methods to evaluate the accuracy of model simulations and constrain parameter distributions [24]. Specifically, we used approximate Bayesian computation (ABC) to evaluate model projections against a multivariate target [50] consisting of (i) correct spatio-temporal occurrence, (ii) persistence in the Caucasus at the end of the simulation and (iii) two persisting refugial populations in 1850 CE. Spatio-temporal occurrence was correct if animals were simulated at a fossil site (and/or its eight nearest neighbouring cells) at the time that the fossil was deposited ±2 × s.d. of the calibrated ^14^C date. For the observation records, there was no temporal band of uncertainty. For simulations that did not result in persistence in the Caucasus Mountain region (as well as Białowieża Forest) at the end of the simulation, we applied an annual penalty for each year that extirpation occurred before 1850 CE. Because European bison had collapsed to two refugia by 1850 CE [3,51], we calculated the difference between the simulated and expected number of populations extant at 1850 CE.

The best 0.25% of feasible parametrizations of European bison-climate-human interactions were identified using the rejection algorithm in the abc package [52]. We then ran three further rounds of POM (each with 10 000 simulations) using informed prior distributions based on these top models [28]. We ceased POM after these three additional rounds, because Bayes factors indicated that the posterior distributions had converged [53]. We did posterior predictive checks on the best 25 (0.25%) of 10 000 models from the final round of POM, using 1000 simulations (based on the posterior distributions of their parameters) and did a goodness-of-fit test with the gfit function [54]. We did two additional independent validation tests of these best SEPMs. Inferences of change in population size (based on N_e_) from ancient DNA (aDNA; electronic supplementary material, methods figure S2) were used to assess whether these models could reconstruct relative change in total population size, assuming that trends in N_e_ and population abundance are positively correlated [6]. Historical sighting records (1000–1500 CE) not used elsewhere in model development or testing were used to test whether the models could reconstruct recent patterns of extirpation. See electronic supplementary material, methods for details.

The best European bison-climate-human interaction models from the final round of simulations were used to generate weighted ensemble averaged estimates of spatial abundance, extirpation time, total population size and harvest rates. Estimates were weighted by the inverse of the Euclidean distance of the model from the validation targets, giving higher weights to models that best reproduced the multivariate target.

### (e) Statistical analysis

We used generalized additive models (GAMs) implemented in the mgcv R package [55] to investigate the drivers of bison abundance in the Pleistocene (21–11.7 ka BP), early-to-mid-Holocene (11.7–4.25 ka BP) and late Holocene (4.25–0.45 ka BP). We extracted total bison abundance at generational time steps from the best SEPMs chosen using POM. The drivers tested were climate (average annual temperature), hunting pressure (human population size) and land use change (human-driven change in vegetation biomass) in occupied grid cells. European bison abundances and the three covariates were aggregated to 100-year time bins to remove the effects of short-term decadal variation and temporal autocorrelation. This approach has been used elsewhere [6].

All GAMs included model ID as a random effect. Fixed effects and any interactions were modelled as penalized thin-plate regression splines. Models were built using a double penalty approach whereby coefficient estimates could be reduced to zero for non-informative covariates [56]. GAMs were optimized using maximum likelihood, with model selection based on a χ^2^ test performed on two times the difference in the minimized smoothing parameter (i.e. maximum likelihood) between GAMs with and without interactions. This approach is preferred over AIC selection methods for models that include random effects [57].

### (f) Model scenarios

We ran counterfactual scenarios to disentangle the spatio-temporal roles that environmental change, human hunting and land use change had on the range collapse of the European bison [28]. We simulated three counterfactual scenarios: *no hunting*, whereby the human hunting rate was set to zero throughout the simulations; *no land use change*, in which biomass of temperate and boreal trees and shrubs was not reduced by land use change; and *no human pressures*, which combined the effects of the *no land use change* and *no hunting* scenarios.

Hunting pressure on European bison increased greatly after 1500 CE, due to technological advancements in hunting and cultural shifts in land use [58], including establishment of royal hunting reserves [51]. To address this, we ran scenarios of increased hunting from 1500 CE to 1870 CE with harvest rates increasing at 10% intervals from 10 to 100% of pre-1500 CE maximum hunting rate. We validated the final abundance in 1870 against a historical estimate of 3560 European bison, with 2000 bison in the Caucasus [51] and 1560 bison in Białowieża Forest [3].

## 3. Results

### (a) Pattern-oriented validation

Our best 25 SEPMs (0.0005% of all models) were able to reconcile inferences of spatio-temporal occurrence and persistence (electronic supplementary material, figure S1). These models correctly reconstructed the timing and place of occurrence of European bison at most fossil sites, and persistence in the Caucasus (and Białowieża Forest) in 1850 CE. The target of two refugial populations of European bison in 1850 CE could not be simulated without increasing hunting after 1500 CE (electronic supplementary material, figure S1; see ‘Drivers of decline' below). Importantly, goodness of fit tests showed no significant difference between the distributions of simulated and inferred summary metrics for all validation targets (*p* > 0.05), indicating a good fit of the simulated to the observed data.

Independent validation tests confirmed that the best SEPMs were robustly parametrized. Confidence intervals of trends in simulated relative abundance overlapped aDNA estimates, with high concordance between simulated and inferred rates of decline in relative population size during the Pleistocene–Holocene transition (electronic supplementary material, figure S2). These best models reconstructed spatio-temporal occurrence at up to 4 of the 5 independent sites of historical occurrence (mean = 3.3, s.d. = 1.11). Goodness-of-fit tests indicated a reasonable resemblance between simulated occurrence and these independent observations of occurrence (*p* > 0.05).

Reconciling inferences of population persistence and extirpation required European bison to have specific demographic and ecological attributes (electronic supplementary material, figure S3). Comparisons of prior and posterior distributions (electronic supplementary material, table S1) show that the European bison is likely to have had a realized niche characterized by a small-to-medium niche volume (58–72% of the volume of the full potential realized niche volume) and medium-to-high specialization (based on niche marginality). These comparisons also showed that a small Allee effect (9 bison per 87 km × 76 km grid cell) and a low maximum dispersal distance (110 km) are needed to reconcile inferences of past population and range dynamics from palaeo and historical archives. In each generation, approximately 5% of bison permanently dispersed, moving at least 76 km from their site of birth. In the best models, maximum harvest was 5–21% of the bison population, with a functional response closer to a type II than type III response (electronic supplementary material, figure S4). The maximum population density in the best models was 0.3 bison km^–^^2^ (approx. 2000 bison per 87 km × 76 km grid cell), which falls within the range of bison population densities measured in Białowieża Forest (0.2–1.5 bison km^–^^2^) [59].

### (b) Population and range dynamics

**Figure 1.**
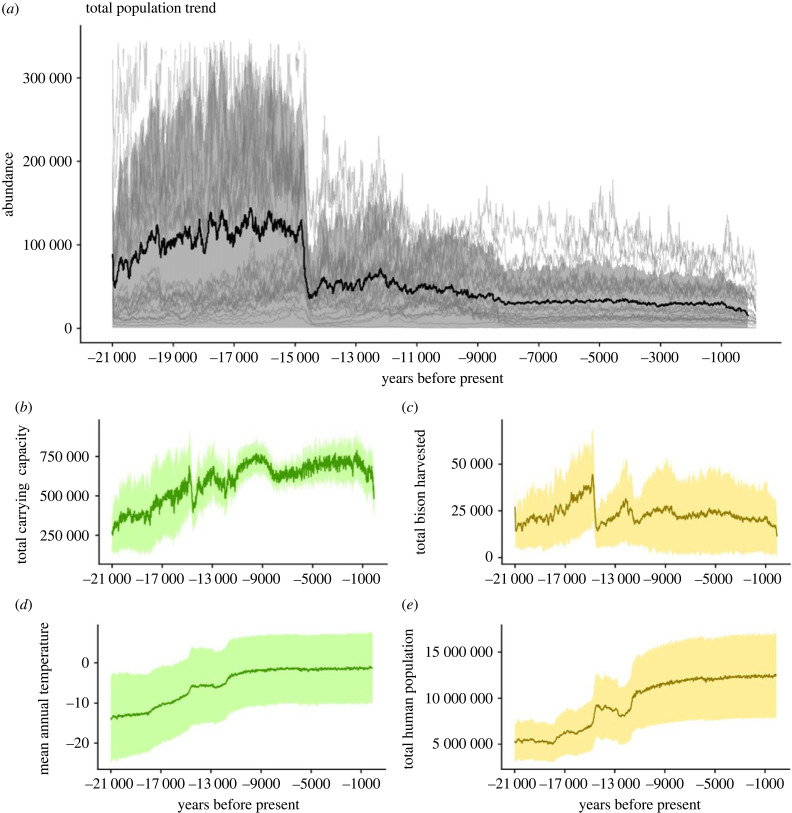
European bison abundance and its drivers projected from 21 000 BP to 450 BP (1500 CE). (*a*) Total European bison population size. The population time series of the best 25 models are shown in light grey. (*b*) Carrying capacity, (*c*) harvested animals, (*d*) mean annual temperature and (*e*) total human population size for the study region. Shading shows ±1 s.d. Carrying capacity represents maximum potential European bison population size in the absence of human impacts. Changes in other climate and environmental variables (including those used to estimate the bison niche) are shown in the electronic supplementary material, methods.

**Figure 2.**
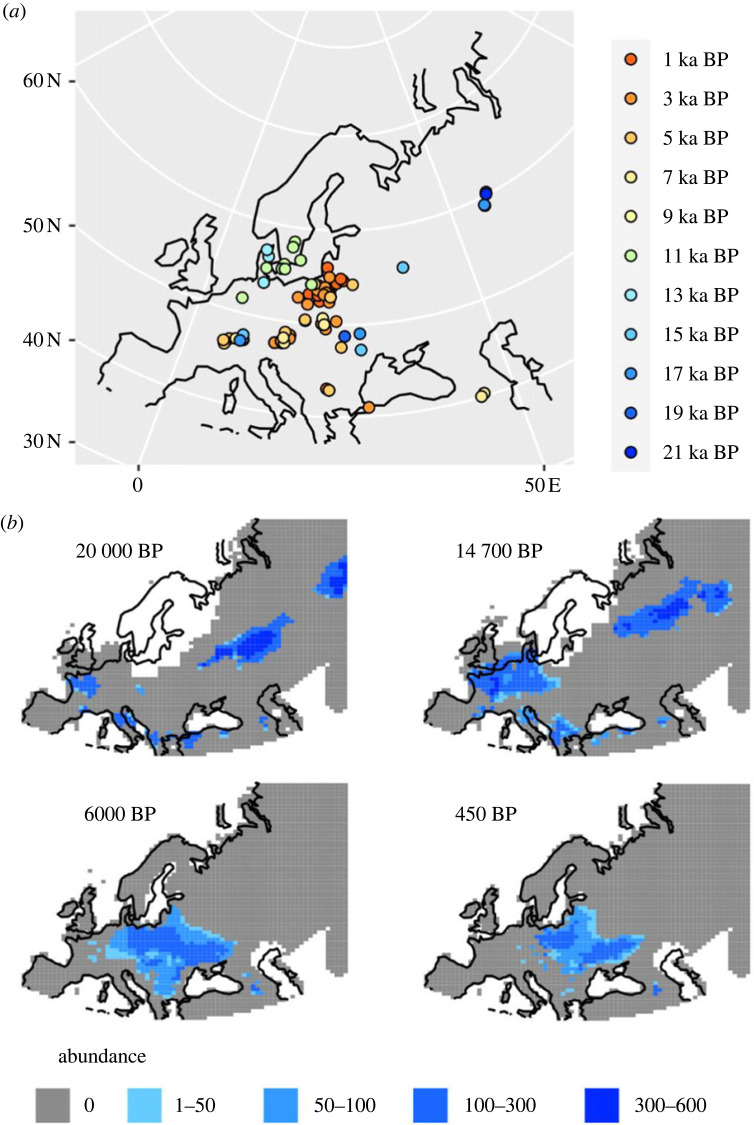
Range collapse of the European bison. (*a*) Fossils and their radiocarbon dates for the European bison. (*b*) Simulated bison abundance for the end of the last glacial maximum stadial (20 000 BP), immediately prior to the Bølling–Allerød warming event (14 700 BP), the mid-Holocene (6000 BP) and at the end of the simulation (450 BP or 1500 CE). Abundances are shown only for grid cells where at least 25% of the top models agreed that there was bison occupancy. See electronic supplementary material, figure S6 for abundance maps that include cells with low model agreement.

Based on the ensemble average of the best SEPMs, the European bison was abundant and widely distributed at the LGM (figures 1 and 2). Population numbers remained relatively stable, increasing slightly during the onset of deglacial warming. They then fell sharply at 14.7 ka BP (Bølling–Allerød warming event), in response to rapid warming and a corresponding reduction in bison carrying capacity. Following 14.7 ka BP, the range-wide carrying capacity of European bison recovered, but abundance did not, probably because of hunting by humans. Hunting of European bison increased after 14.7 ka BP due to increased human abundance, which remained high throughout the remainder of the simulation (figure 1).

Reconstructions of spatio-temporal abundance of the European bison show that by the mid-Holocene it had contracted its range to central and eastern Europe and the Caucasus, going extinct in southern Europe at approximately 11 ka BP, and in Western Europe at approximately 7 ka BP (figure 2; electronic supplementary material, movie S1). During the early deglaciation (21–18 ka BP), European bison were distributed in disjunct metapopulations in Siberia, the Caucasus, southern Europe and western Europe. From 18 ka BP, metapopulations in western and southern Europe started to slowly move eastward and northward, merging together some 6000 years later (electronic supplementary material, movie S1). By 12 ka BP, the only remaining European bison in Western Asia were in the refugium in the Caucasus. The Siberian metapopulation declined in size from 13 ka BP, going regionally extinct at 8 ka BP. By 1500 CE (or 450 BP), the European bison was restricted to north-eastern Europe and a small refugium in the Caucasus, attaining highest abundances in the Caucasus and in what is now Poland and Ukraine (figure 2).

### (c) Drivers of decline

**Figure 3.**
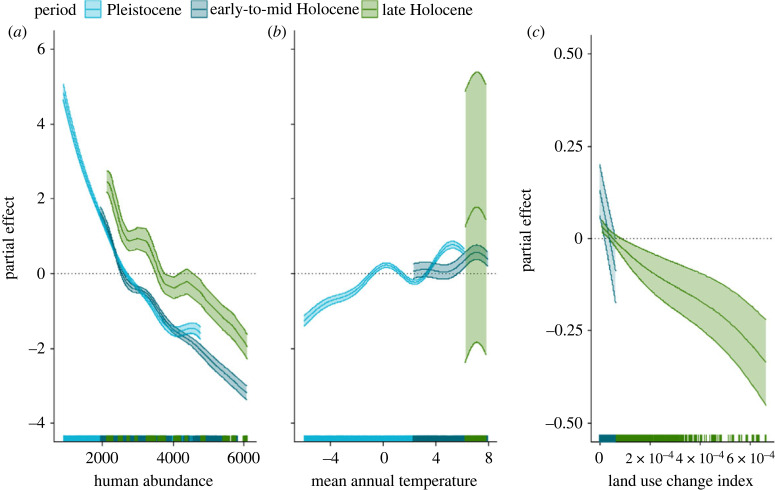
Predictors of population decline in European bison. Partial effects plots for general additive models of drivers of bison abundance with respect to (*a*) human abundance, (*b*) mean annual temperature and (*c*) land use change. Different colours show different time periods: Pleistocene (light blue), early-to-mid-Holocene (dark blue) and late Holocene (green). Rug plots of sampling density are shown along the *x*-axis.

Analysis of total population size of European bison from our best SEPMs showed that hunting pressure was the primary determinant of population decline during the Pleistocene, early-to-mid-Holocene and late Holocene (figure 3). The GAM of bison abundance regressed against mean annual temperature, human abundance and land use change explained 89% of variance in total population size (adjusted *R*^2^ = 0.83). This was a significant improvement over the same model with interactions between variables (delta maximum likelihood = 123.13, df = 12, *p* < 0.001). A strong positive correlation between temperature and European bison abundance was detected during the Pleistocene, with a weakly positive effect in the early-mid-Holocene. The effect of land use change on vegetation biomass had a slight negative effect on bison abundance in the Holocene (electronic supplementary material, table S2).

**Figure 4.**
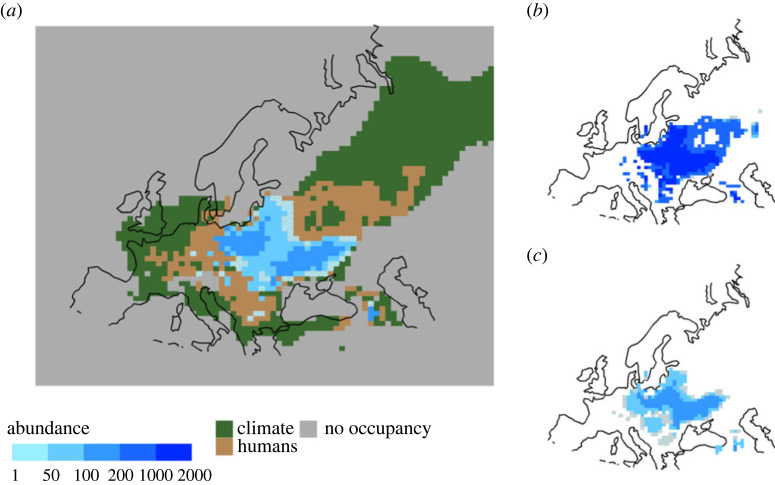
Drivers of range collapse for European bison. (*a*) The effect of climate (green) and humans (brown) on the extirpation of European bison. Population abundance for the projected extant range in 1500 CE is shown in blue. (*b*,*c*) Maps of abundance of European bison are shown in 1500 CE without hunting (*b*) and without land use change (*c*). Grid cells are 86.6 by 75.6 km. Abundances are shown only for grid cells where at least 25% of models in the baseline scenario agreed that there was bison occupancy. Hatched areas indicate areas of very low abundance (<50 bison).

Counterfactual scenarios revealed the spatio-temporal footprint of humans on the local persistence of European bison (figure 4). In the absence of hunting and land conversion by humans, European bison are likely to have persisted much longer in Scandinavia, the Balkans, and present-day Germany and southwestern Russia (electronic supplementary material, figure S5). They also show that climatic change during the Pleistocene was the primary determinant of range contractions in Western Europe, Anatolia and Siberia (figure 4). During the Holocene, hunting caused range loss in the north and east of the European bison distribution, while change in land use was responsible for losses in the west and south.

In the *no hunting* scenario, the range of the European bison in 1500 CE extended farther east into Russia, while the Caucasus refugium (where the European bison persisted until its extinction in the wild) extended farther north (figure 4). In the *no land use change* scenario, the European bison's range extended farther west in both continental Europe and the Caucasus (figure 4). In the *no human pressure* scenario, there were additional areas in southern Denmark and France where the removal of both hunting and land use change allowed European bison to persist to 1500 CE. A rasterized map of causes of local extinctions of European bison populations based on these counterfactual scenarios is provided [60].

**Figure 5.**
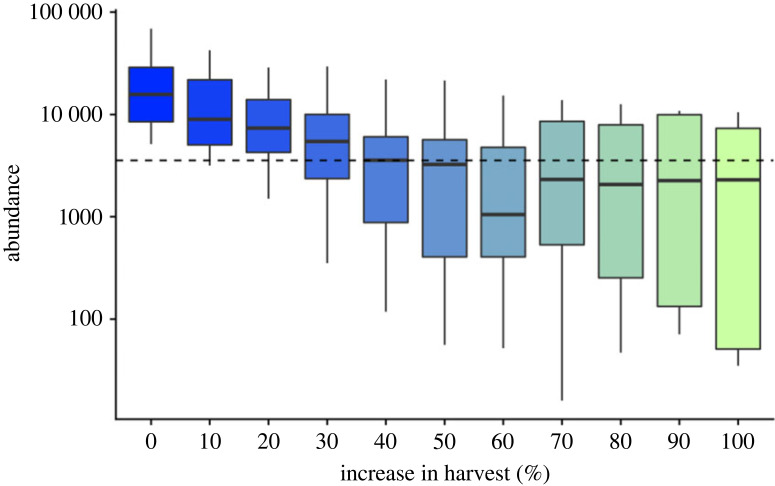
Harvest increase needed to simulate population size in 1870 CE. Population size of European bison in 1870 CE in response to increased harvesting following 1500 CE. Dashed horizontal line shows estimated population size of European bison in 1870 CE [3,51]. Estimates are based on the output from individual runs of the best 0.25% of process-explicit models.

While our best SEPMs overshot the estimated population size of European bison in 1870 CE (*n* = 3560) (figure 1), a 30% increase in harvest rate after 1500 CE (in response to cultural and technological changes in hunting, including guns [61]) was enough to deplete the population size to levels observed in 1870 CE (figure 5).

## 4. Discussion

Using spatially explicit process-driven ecological models that integrate extensive palaeontological and historical data, including new radiocarbon-dated fossils, we were able to disentangle the spatio-temporal effects of climatic change, human hunting and land use change on the population and range dynamics of European bison following the last ice age. We show that the effects of natural and anthropogenic drivers varied from the edge to the core of the European bison's distribution. Humans had the most pronounced impact in the centre of its distribution, contributing heavily to the population decline and range collapse of the European bison, while climatic change is likely to have caused losses of peripheral populations. By identifying areas where the European bison would have persisted to 1500 CE in the absence of human pressures, we pinpoint locations that are potentially climatically suitable for reintroduction of European bison today.

Our high-resolution reconstruction of the range and population dynamics of the European bison shows that its persistence was affected by biotic and abiotic stressors that varied spatio-temporally. Increases in temperature had a strong positive impact on European bison abundance, but only in the Pleistocene, with subsequent warming having a reduced impact. We show that environmental change in the late Pleistocene caused the range of the European bison to shift from a fragmented periphery to a centralized core population in Europe where human abundances in Eurasia were generally highest [40]. Here, European bison were hunted for food and skins [62], and for noble prestige from the late medieval period [63]. Reconciling inferences of demographic change from the extensive fossil record of European bison required process-explicit models to have a medium level of sustained hunting by humans (5–21% maximum harvest rate for European bison). These rates align with isotopic evidence from human fossils from Europe during the late Pleistocene, which suggest that 10% of average protein intake came from bovines (aurochs and European bison) [64]. Increasing geographical overlap between areas of high bison abundance and high human abundance, along with limited dispersal between increasingly isolated fragments of populations of European bison, eventually led to the demise of the species in the wild, both directly through overexploitation by hunting, and indirectly through land use change, reducing abundances to a level where mean individual fitness is likely to have declined.

The abundance of European bison today is trending upward following nearly 100 years of conservation intervention [3]. However, the species still faces many threats and obstacles to its long-term persistence [4]. Contemporary threats include land use changes that reduce carrying capacity and impose barriers to dispersing bison through fragmentation [65], and poaching that reduces population numbers [4,18]. These are the very same threats that put the European bison on a pathway to extinction many millennia ago, as shown by our validated models.

As a priority species for conservation in Europe [66] because of its important role in restoring grassland habitat [67], a lot of money and time is being invested into expanding the range and abundance of European bison [4], but with mixed success. Of the 47 free-living European bison populations, only eight have more than 150 adults, and all eight of these are dependent on supplemental feeding due to poor habitat suitability [4]. Moreover, only 23% of reintroduced populations of European bison have connectivity to other populations [4]. Managers clearly need new information that will help to maximize the probability of successful reintroductions of European bison, and potential flow-on benefits to nature and people [16]. This information includes, but is not limited to, knowledge of the geographical structure and dynamics of the range of the European bison immediately prior to and during its demographic decline [7]. Without this biogeographic information, the consequences of improper management decisions can be severe. Reintroductions of European bison into unsuitable areas have already failed or experienced serious problems due to slow-growing populations succumbing to inbreeding depression (e.g. in Siberia [68]).

Our spatial reconstructions of European bison abundance in 1500 CE provide critical new information to guide European bison reintroductions. Poland, areas of Ukraine and areas bordering western Russia are areas of highest projected abundance in the fifteenth century. Today, Ukraine and western Russia are characterized by widespread abandonment of agricultural land [69], making them very suitable for reintroductions of European bison. Indeed, over 50% of all free-living European bison are found in these regions [18], which is unfortunate given that it is currently an active conflict zone. However, our modelling also shows that there are other regions that are likely to have had medium-to-high densities of European bison until at least 1500 CE, even with sustained levels of hunting. These include Slovakia, Romania and the Caucasus.

Maps of abundances of European bison in 1500 CE in the absence of human pressures reveal additional potential regions and sites for future reintroduction and habitat restoration [60]. For example, we show that land use change is likely to have contributed to the extirpation of European bison in Germany, Czechia, Bulgaria and Serbia during the late Holocene, indicating that these areas are also likely to provide suitable current-day reintroduction sites if adequate habitat restoration is done. These counterfactual simulations also reveal areas that have been climatically unsuitable for European bison for many millennia and thus should be excluded from current translocation and reintroduction efforts. Looking ahead, our process-explicit models of European bison–climate–human interactions could be used to identify reintroduction sites that are not only suitable for conservation management today, but also under future anthropogenic climate and environmental change.

Our reconstructions of the range and population dynamics of the European bison also offer new and important biogeographical insights. We provide evidence for an isolated metapopulation of European bison in Siberia until 7 ka BP that went extinct due to deglacial environmental change. This finding is consistent with inferences from ancient DNA of a cold-adapted clade of European bison that went extinct during the most recent deglaciation [1]. Our validated modelling suggests that this clade is likely to have survived for longer than previously thought, persisting into the Holocene in low abundances. We also show that in Europe at 21 ka BP there were two main subpopulations of European bison, one in Western Europe and one in Southern Europe, and these subpopulations fused together in Central Europe at the termination of the Pleistocene. Population genomics could be done to further verify this finding [70].

Our validated simulation models show that while human pressures were a major contributor to the demographic and range collapse of the European bison during the Pleistocene–Holocene transition, environmental change was also needed to cause its demise. Our findings rule out the hypothesis that habitat loss due to deforestation was the primary cause of the range contractions and near extinction of the European bison, because the effect of land use change on bison abundance was minor compared to mean annual temperature and human hunting. Our results do not, however, rule out the theory that European bison were primarily grazers that were forced by environmental change and human pressures into suboptimal forest habitat. They cannot definitively confirm it either, because the spatio-temporal resolution of our data on palaeo-vegetation was not fine enough to conclusively identify European bison preferences for open or closed habitat.

While our simulation approach was able to reconstruct many key features of the range dynamics of the European bison over the last 21 000 years, the approach overestimated its population size in 1870 CE. This is likely because of a technological and cultural shift in bison hunting after 1500 CE [61]. Indeed, we show that a 30% increase in harvest efficiency after 1500 CE was enough to reconstruct the population size of 3560 animals estimated in 1870 CE. Firearms began to be banned by some European governments in the sixteenth century in order to preserve game populations due to overhunting with this new type of weaponry [71]. We also found that reconstructing our validation targets required a relatively short maximum dispersal distance for European bison. Dispersal in our models represents permanent relocations to new breeding territory, rather than the temporary movements of individuals. While research on European bison dispersal in Białowieza Forest has found that cow groups (equivalent to what was modelled here) disperse much shorter distances than individual males [3], further tests of the dispersal estimates that emerged from our model are needed. We also observed high variance among the selected models in our validated ensemble in their estimates of bison population size before 15 ka BP. The precision in this estimate would be improved by more refined validation targets, particularly in estimates of demographic change from Pleistocene fossils and ancient DNA.

By reconstructing past abundances and harvest rates, as well as mapping the distribution of the European bison in the absence of human pressures, we were able to decipher the millennial processes of population decline, range contraction, and near extinction of the European bison, providing data and models needed to improve the on-ground conservation and rewilding of European bison. New insights into the biogeography of the species were made possible because our validated process-driven ecological modelling framework allowed us to reconstruct its abundance as well as its likelihood of occurrence. Similar approaches could be used to reconstruct the causes of population declines and range collapses of other large herbivores being reintroduced to Eurasia [6] and other continents [7], including American bison (*Bison bison*), improving awareness of past threats and enriching current conservation actions.

## Data Availability

Data and code are accessible on Figshare and Zenodo: https://doi.org/10.5281/zenodo.7297867/ [26], https://doi.org/10.6084/m9.figshare.23688981.v1 [60], https://doi.org/10.6084/m9.figshare.21624369.v1 [72] and https://doi.org/10.6084/m9.figshare.21521190.v2 [25]. Supplementary material is available online [73].
